# Supplementary material for: Ubiquitination Occurs in the Mitochondrial Matrix by Eclipsed Targeted Components of the Ubiquitination Machinery
Source: Cells. 2022 Dec 17;11(24):4109. doi: 10.3390/cells11244109 (PMC9777009; doi:10.3390/cells11244109)
Supplement: Supplementary file 1 [file cells-11-04109-s001.zip › cells-2034405-supplementary.pptx]

## Slide 1
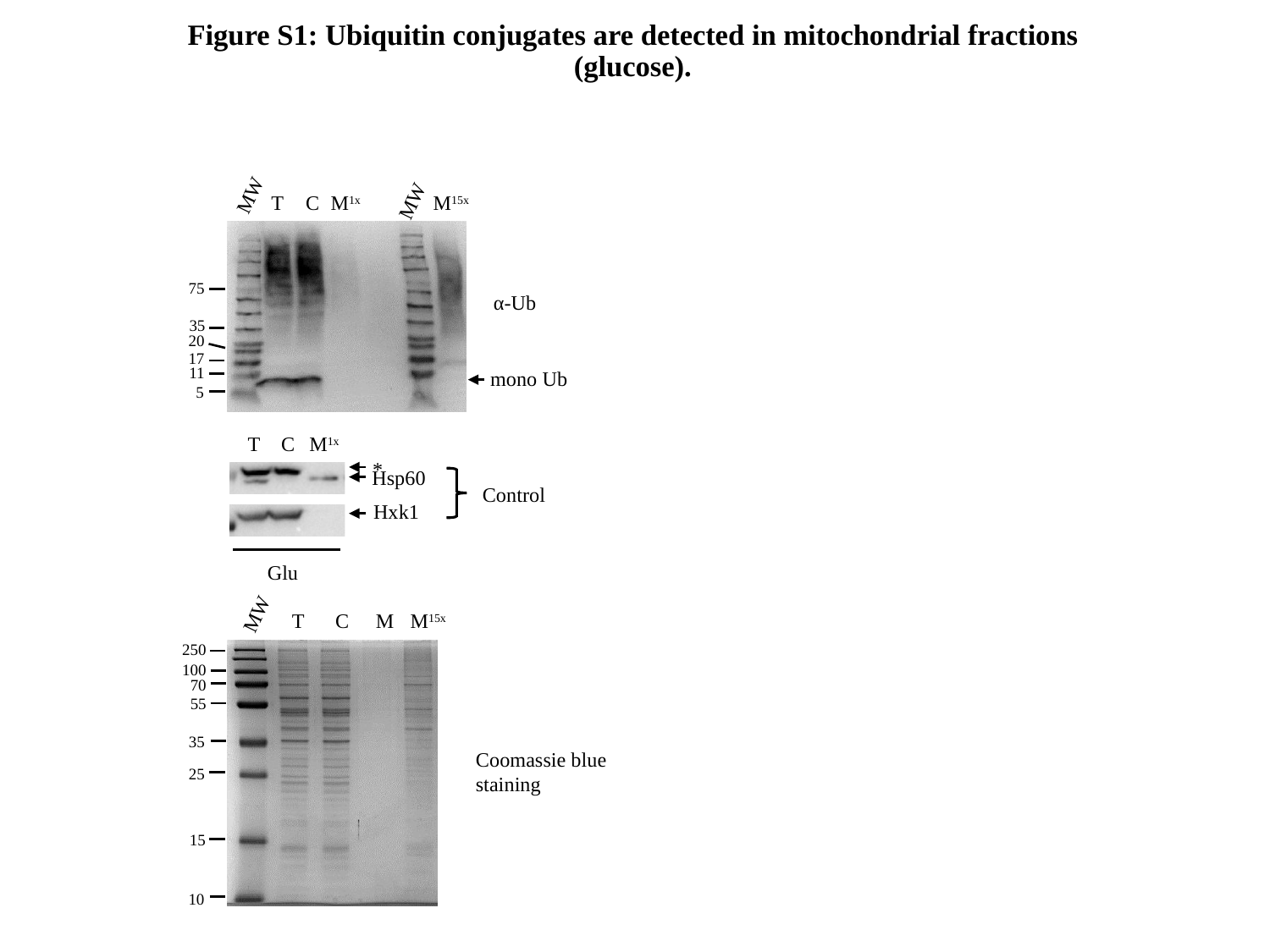

# Figure S1: Ubiquitin conjugates are detected in mitochondrial fractions (glucose).
MW
MW
M1x
T
C
M15x
75
35
20
17
11
5
 α-Ub
mono Ub
M1x
T
C
Glu
 *
Hsp60
 Control
Hxk1
MW
C
T
M15x
M
250
70
55
25
15
10
100
35
Coomassie blue staining

## Slide 2
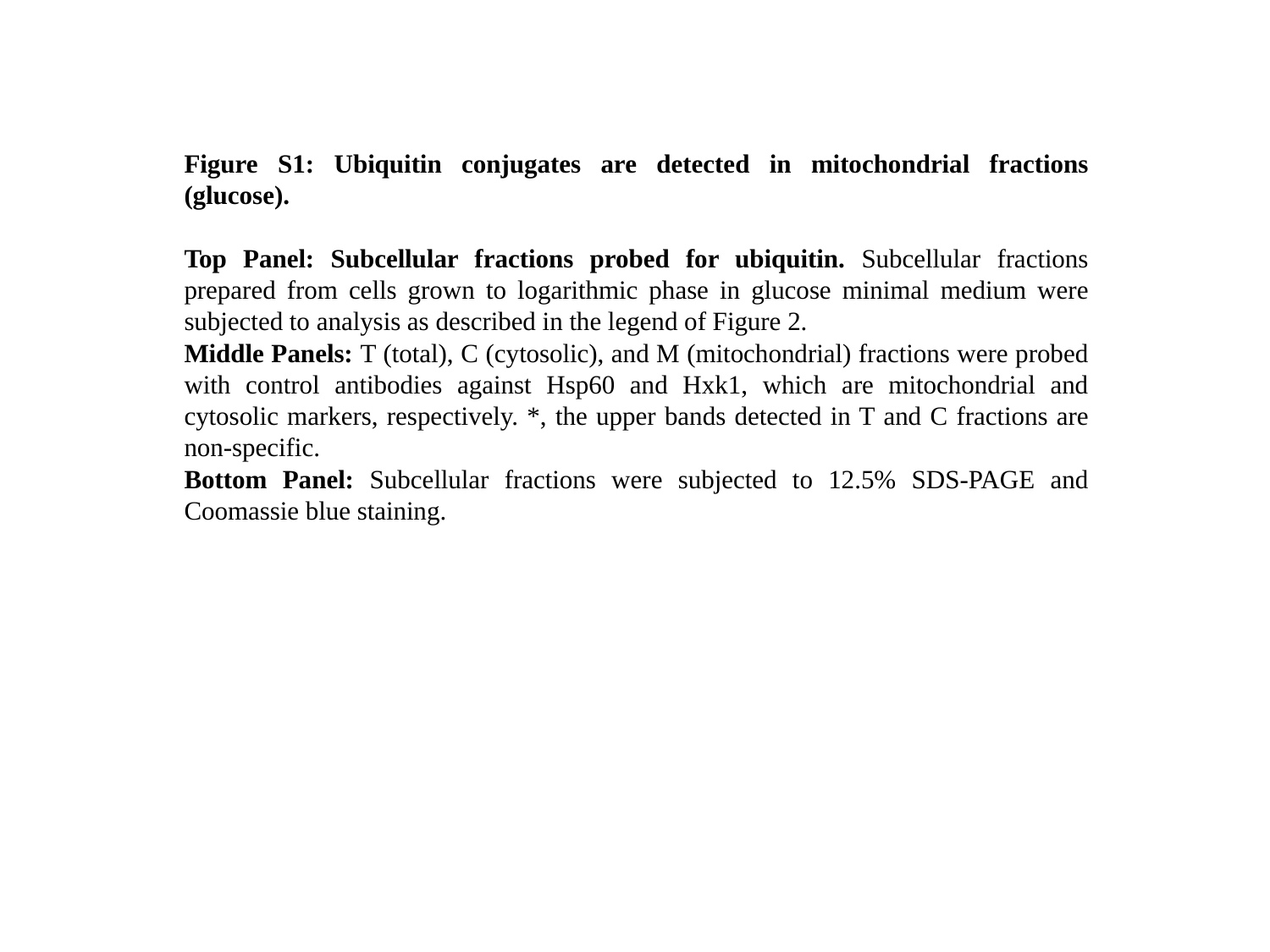

Figure S1: Ubiquitin conjugates are detected in mitochondrial fractions (glucose).
Top Panel: Subcellular fractions probed for ubiquitin. Subcellular fractions prepared from cells grown to logarithmic phase in glucose minimal medium were subjected to analysis as described in the legend of Figure 2.
Middle Panels: T (total), C (cytosolic), and M (mitochondrial) fractions were probed with control antibodies against Hsp60 and Hxk1, which are mitochondrial and cytosolic markers, respectively. *, the upper bands detected in T and C fractions are non-specific.
Bottom Panel: Subcellular fractions were subjected to 12.5% SDS-PAGE and Coomassie blue staining.

## Slide 3
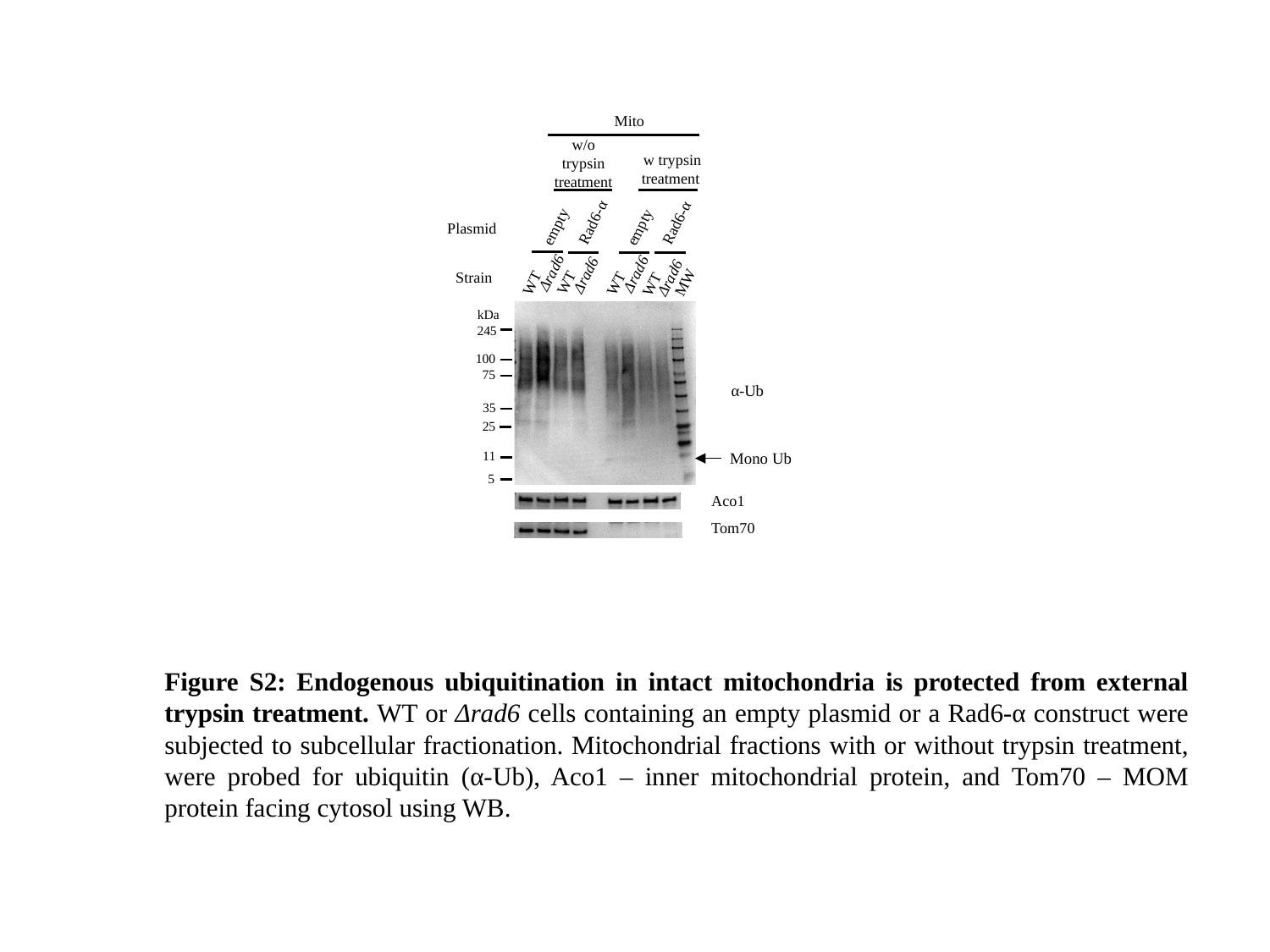

Mito
w/o trypsin
treatment
w trypsin
treatment
 Rad6-α
 Rad6-α
 empty
 empty
Plasmid
Δrad6
Δrad6
Δrad6
Δrad6
WT
WT
WT
Strain
MW
WT
kDa
245
100
75
35
25
11
5
α-Ub
Mono Ub
Aco1
Tom70
Figure S2: Endogenous ubiquitination in intact mitochondria is protected from external trypsin treatment. WT or Δrad6 cells containing an empty plasmid or a Rad6-α construct were subjected to subcellular fractionation. Mitochondrial fractions with or without trypsin treatment, were probed for ubiquitin (α-Ub), Aco1 – inner mitochondrial protein, and Tom70 – MOM protein facing cytosol using WB.

## Slide 4
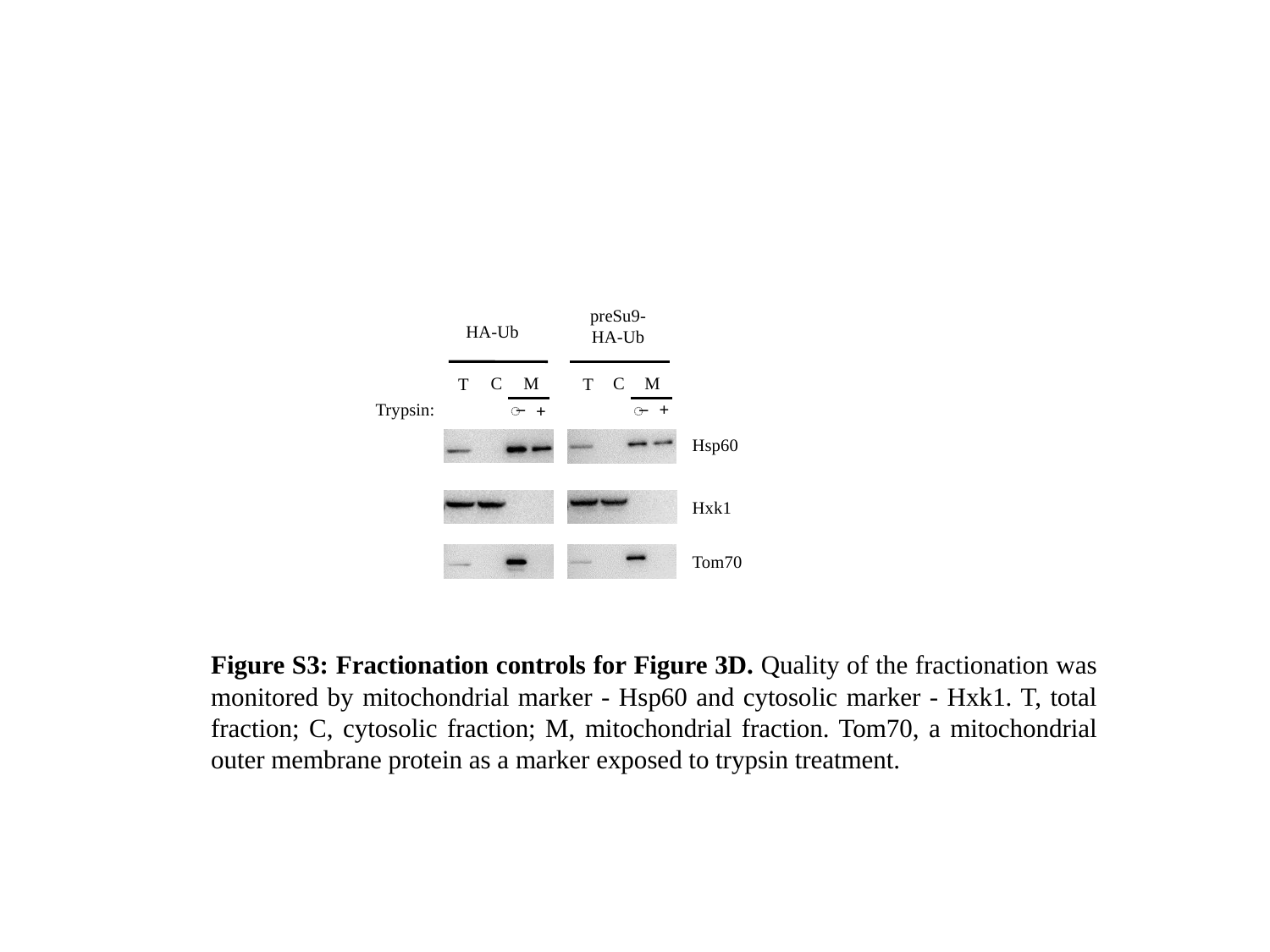

#
preSu9-
HA-Ub
HA-Ub
C
M
T
C
M
T
Trypsin:
+
̶
̶
+
Hsp60
Hxk1
Tom70
Figure S3: Fractionation controls for Figure 3D. Quality of the fractionation was monitored by mitochondrial marker - Hsp60 and cytosolic marker - Hxk1. T, total fraction; C, cytosolic fraction; M, mitochondrial fraction. Tom70, a mitochondrial outer membrane protein as a marker exposed to trypsin treatment.

## Slide 5
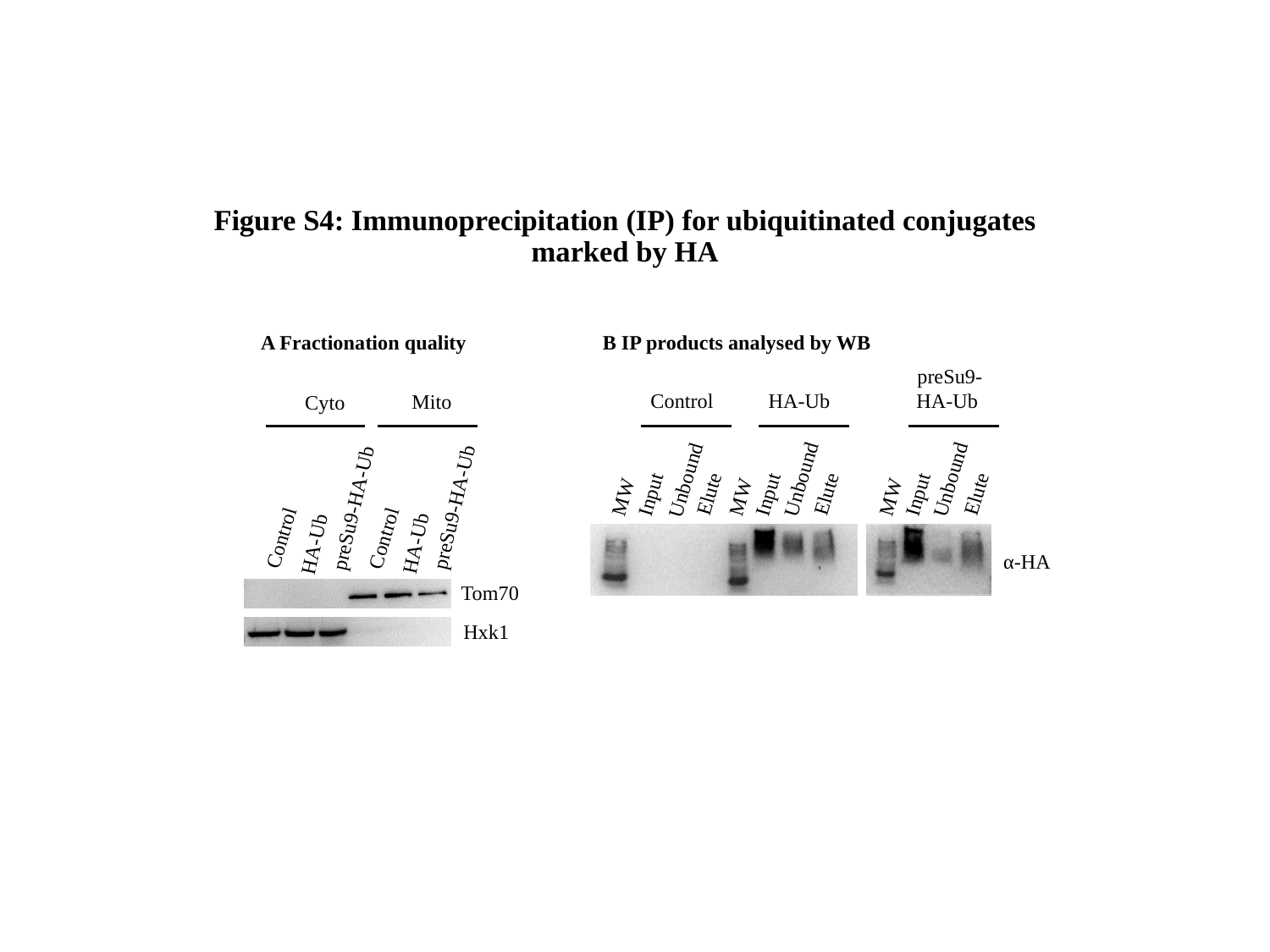

# Figure S4: Immunoprecipitation (IP) for ubiquitinated conjugates marked by HA
A Fractionation quality
B IP products analysed by WB
preSu9-
HA-Ub
Unbound
Input
Elute
MW
Mito
preSu9-HA-Ub
Control
HA-Ub
Cyto
preSu9-HA-Ub
Control
HA-Ub
Control
Unbound
Input
Elute
MW
HA-Ub
Unbound
Input
Elute
MW
α-HA
Tom70
Hxk1

## Slide 6
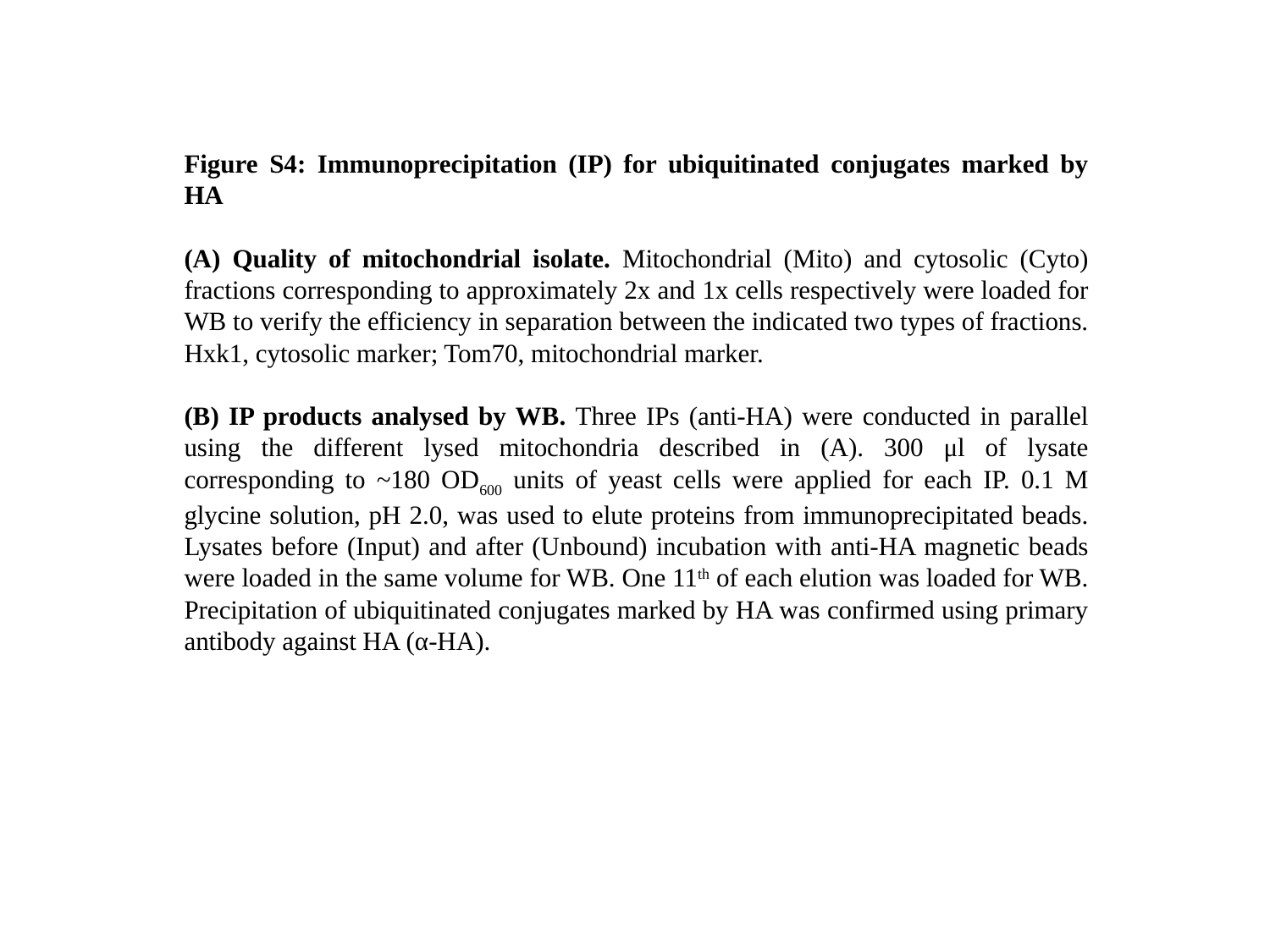

Figure S4: Immunoprecipitation (IP) for ubiquitinated conjugates marked by HA
 Quality of mitochondrial isolate. Mitochondrial (Mito) and cytosolic (Cyto) fractions corresponding to approximately 2x and 1x cells respectively were loaded for WB to verify the efficiency in separation between the indicated two types of fractions. Hxk1, cytosolic marker; Tom70, mitochondrial marker.
 IP products analysed by WB. Three IPs (anti-HA) were conducted in parallel using the different lysed mitochondria described in (A). 300 μl of lysate corresponding to ~180 OD600 units of yeast cells were applied for each IP. 0.1 M glycine solution, pH 2.0, was used to elute proteins from immunoprecipitated beads. Lysates before (Input) and after (Unbound) incubation with anti-HA magnetic beads were loaded in the same volume for WB. One 11th of each elution was loaded for WB. Precipitation of ubiquitinated conjugates marked by HA was confirmed using primary antibody against HA (α-HA).

## Slide 7
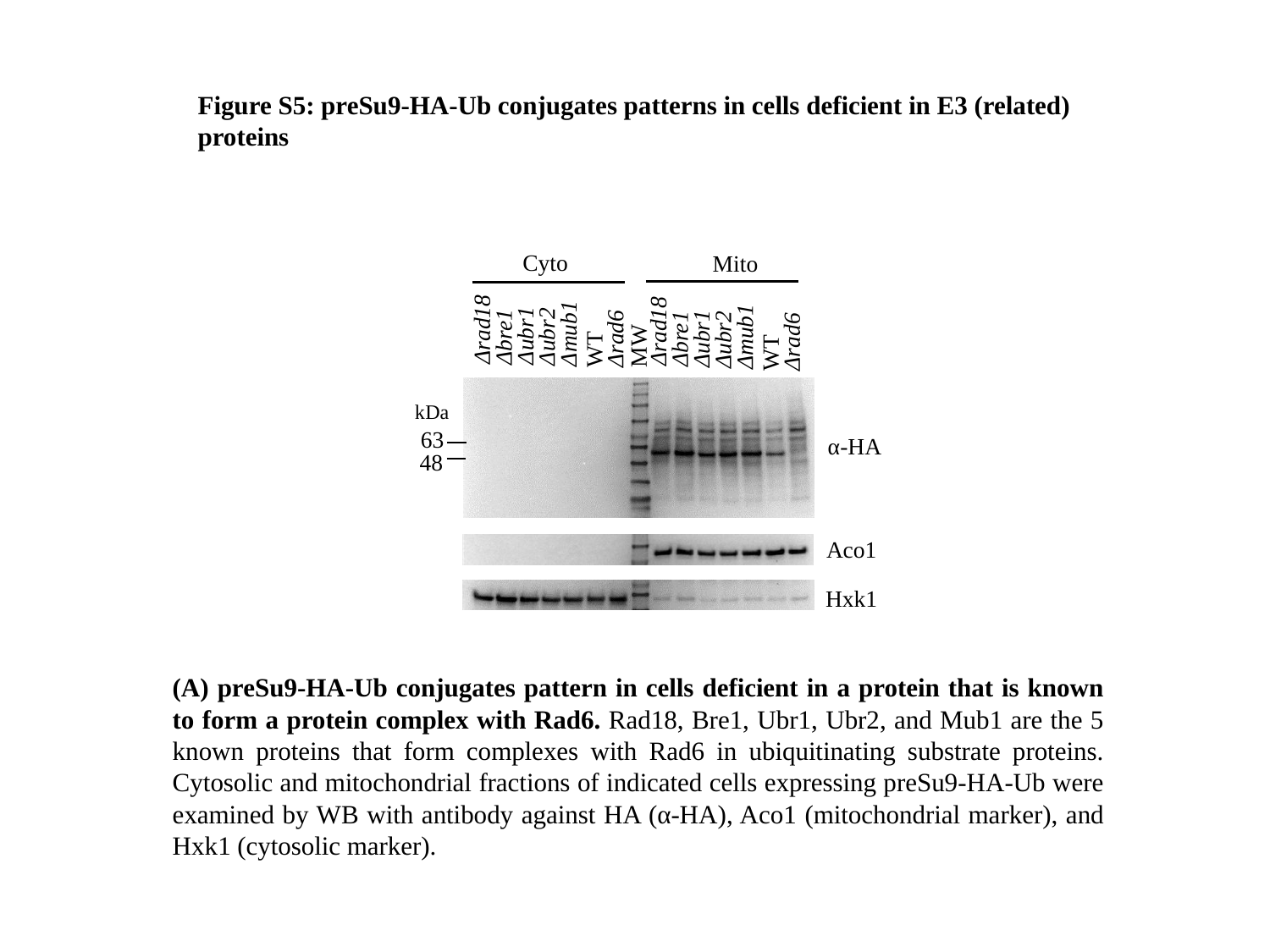

Figure S5: preSu9-HA-Ub conjugates patterns in cells deficient in E3 (related) proteins
Cyto
Mito
Δrad18
Δubr1
Δbre1
Δubr2
Δrad18
Δmub1
Δbre1
WT
MW
Δrad6
Δubr1
Δubr2
Δmub1
WT
Δrad6
kDa
63
α-HA
48
Aco1
Hxk1
(A) preSu9-HA-Ub conjugates pattern in cells deficient in a protein that is known to form a protein complex with Rad6. Rad18, Bre1, Ubr1, Ubr2, and Mub1 are the 5 known proteins that form complexes with Rad6 in ubiquitinating substrate proteins. Cytosolic and mitochondrial fractions of indicated cells expressing preSu9-HA-Ub were examined by WB with antibody against HA (α-HA), Aco1 (mitochondrial marker), and Hxk1 (cytosolic marker).

## Slide 8
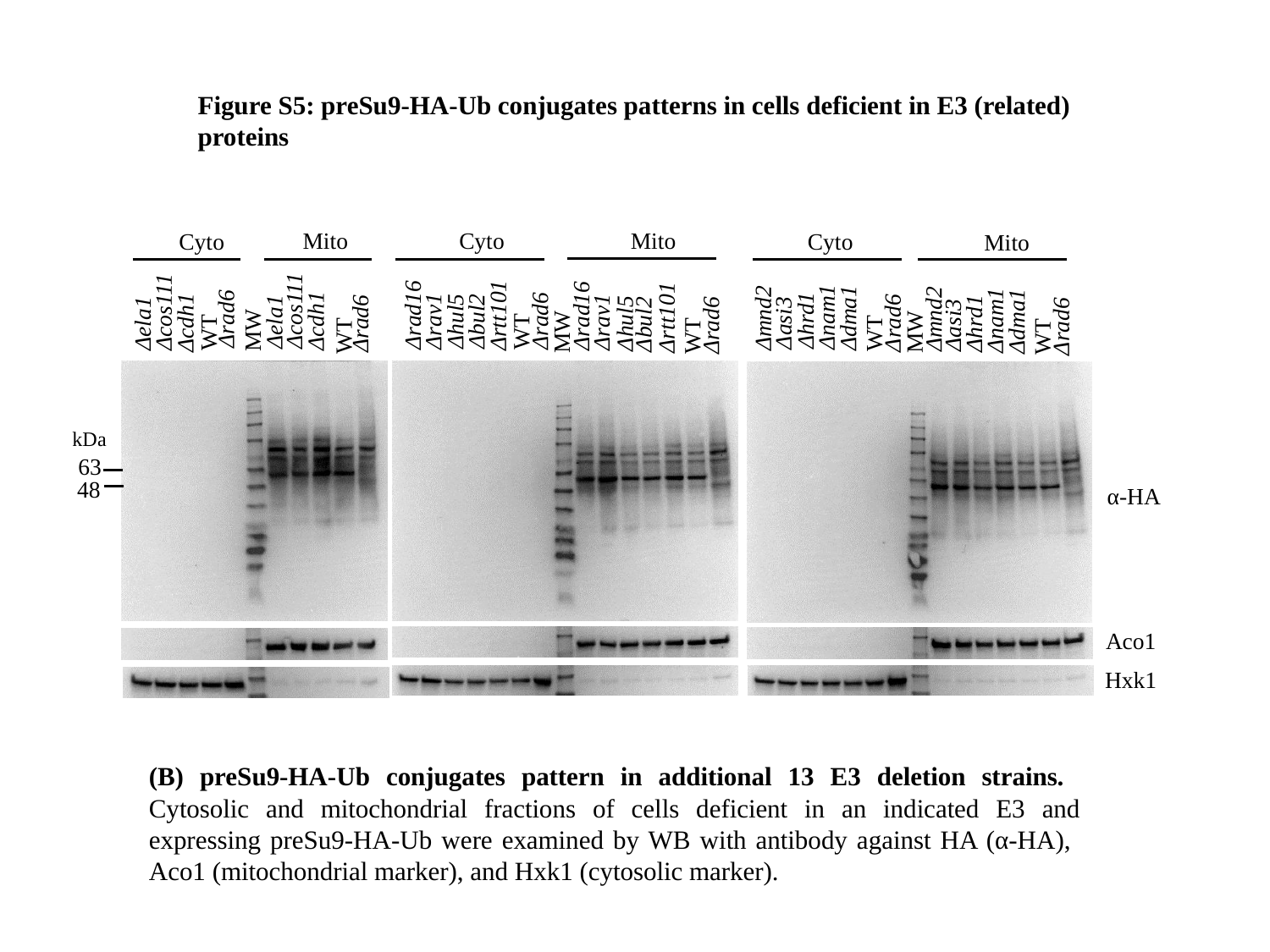

Figure S5: preSu9-HA-Ub conjugates patterns in cells deficient in E3 (related) proteins
Cyto
Mito
Mito
Cyto
Cyto
Mito
Δasi3
Δrav1
Δrav1
Δcos111
Δdma1
Δmnd2
Δrtt101
Δcdh1
Δcos111
Δasi3
Δcdh1
Δrtt101
Δdma1
Δrad6
Δbul2
Δhrd1
Δhul5
Δela1
WT
Δnam1
Δrad16
Δrad6
Δrad16
Δela1
WT
Δhul5
MW
Δmnd2
WT
Δbul2
Δrad6
Δhrd1
Δrad6
MW
Δnam1
MW
WT
WT
Δrad6
WT
Δrad6
kDa
63
48
α-HA
Aco1
Hxk1
(B) preSu9-HA-Ub conjugates pattern in additional 13 E3 deletion strains. Cytosolic and mitochondrial fractions of cells deficient in an indicated E3 and expressing preSu9-HA-Ub were examined by WB with antibody against HA (α-HA), Aco1 (mitochondrial marker), and Hxk1 (cytosolic marker).

## Slide 9
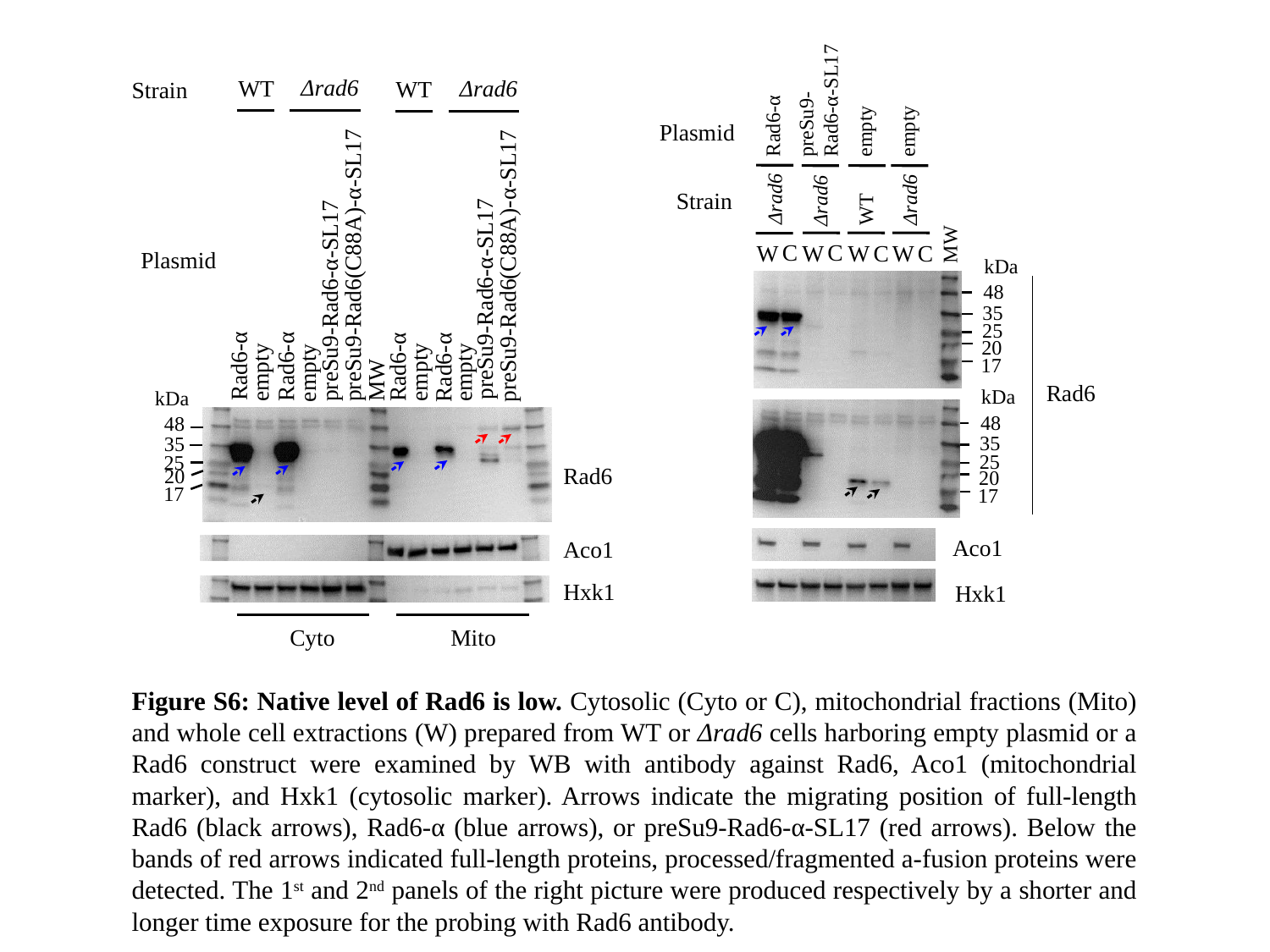

preSu9-
Rad6-α-SL17
Δrad6
WT
Δrad6
WT
Strain
Rad6-α
empty
empty
Plasmid
Strain
Δrad6
Δrad6
WT
Δrad6
MW
C
C
W
C
W
W
C
W
preSu9-Rad6(C88A)-α-SL17
preSu9-Rad6(C88A)-α-SL17
Plasmid
kDa
48
35
25
20
17
preSu9-Rad6-α-SL17
preSu9-Rad6-α-SL17
Rad6-α
Rad6-α
empty
empty
Rad6-α
empty
Rad6-α
empty
MW
Rad6
kDa
48
35
25
20
17
kDa
48
35
25
20
17
Rad6
Aco1
Aco1
Hxk1
Hxk1
Cyto
Mito
Figure S6: Native level of Rad6 is low. Cytosolic (Cyto or C), mitochondrial fractions (Mito) and whole cell extractions (W) prepared from WT or Δrad6 cells harboring empty plasmid or a Rad6 construct were examined by WB with antibody against Rad6, Aco1 (mitochondrial marker), and Hxk1 (cytosolic marker). Arrows indicate the migrating position of full-length Rad6 (black arrows), Rad6-α (blue arrows), or preSu9-Rad6-α-SL17 (red arrows). Below the bands of red arrows indicated full-length proteins, processed/fragmented a-fusion proteins were detected. The 1st and 2nd panels of the right picture were produced respectively by a shorter and longer time exposure for the probing with Rad6 antibody.

## Slide 10
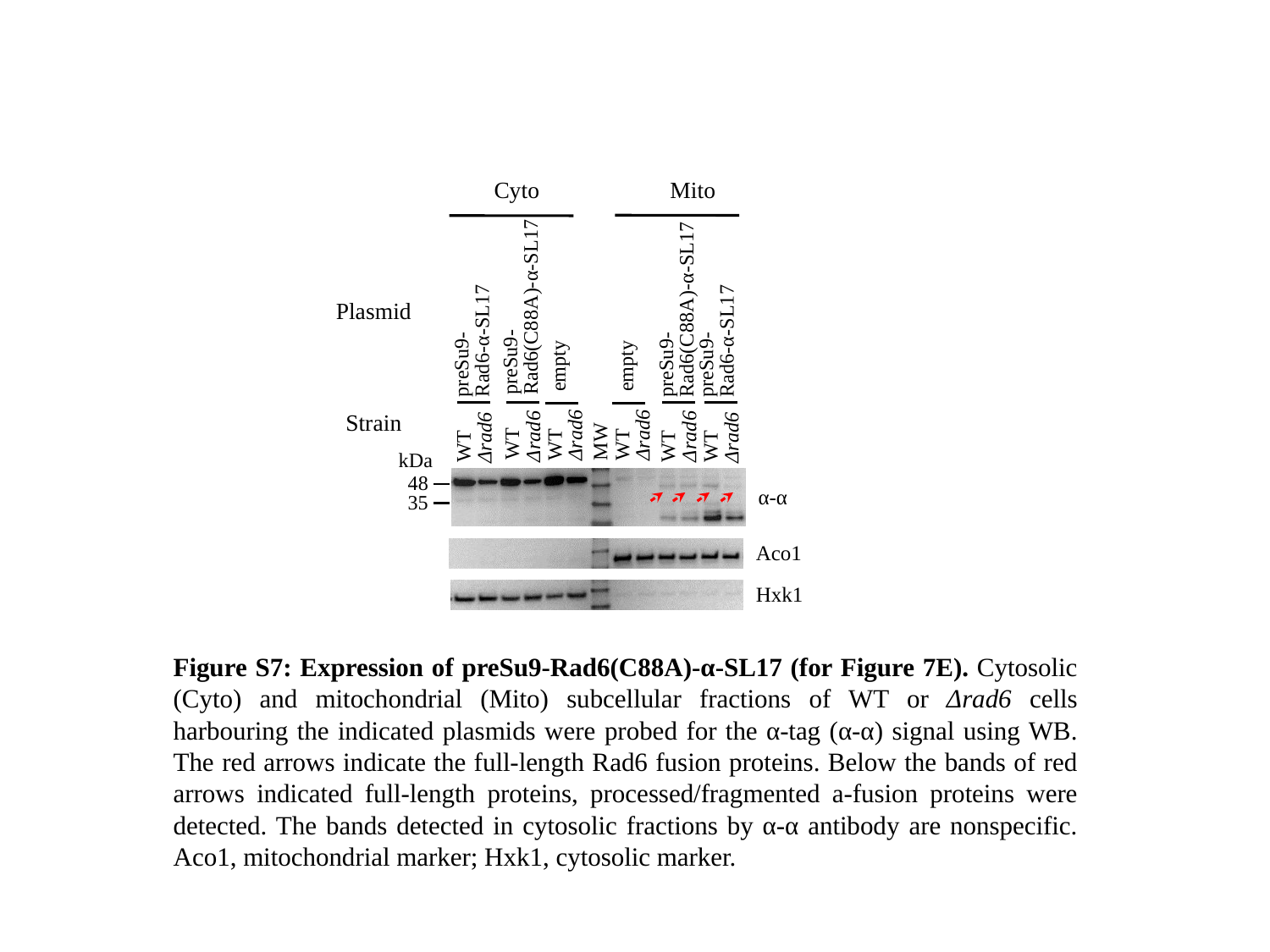

Mito
Cyto
preSu9-Rad6(C88A)-α-SL17
preSu9-Rad6(C88A)-α-SL17
Plasmid
preSu9-Rad6-α-SL17
preSu9-Rad6-α-SL17
empty
empty
Strain
MW
WT
Δrad6
WT
Δrad6
Δrad6
Δrad6
Δrad6
Δrad6
WT
WT
WT
WT
kDa
48
α-α
35
Aco1
Hxk1
Figure S7: Expression of preSu9-Rad6(C88A)-α-SL17 (for Figure 7E). Cytosolic (Cyto) and mitochondrial (Mito) subcellular fractions of WT or Δrad6 cells harbouring the indicated plasmids were probed for the α-tag (α-α) signal using WB. The red arrows indicate the full-length Rad6 fusion proteins. Below the bands of red arrows indicated full-length proteins, processed/fragmented a-fusion proteins were detected. The bands detected in cytosolic fractions by α-α antibody are nonspecific. Aco1, mitochondrial marker; Hxk1, cytosolic marker.

## Slide 11
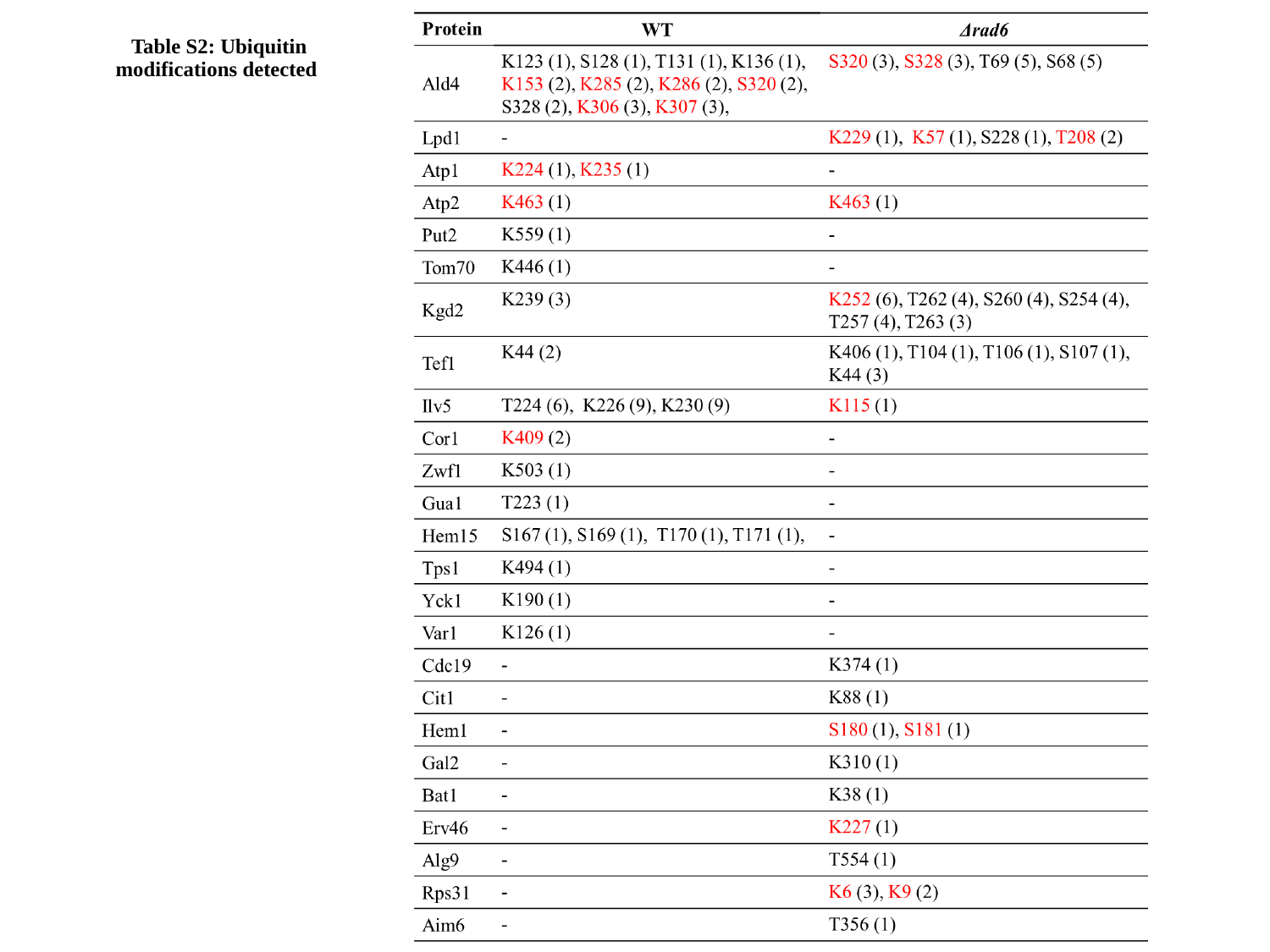

# Table S2: Ubiquitin modifications detected

## Slide 12
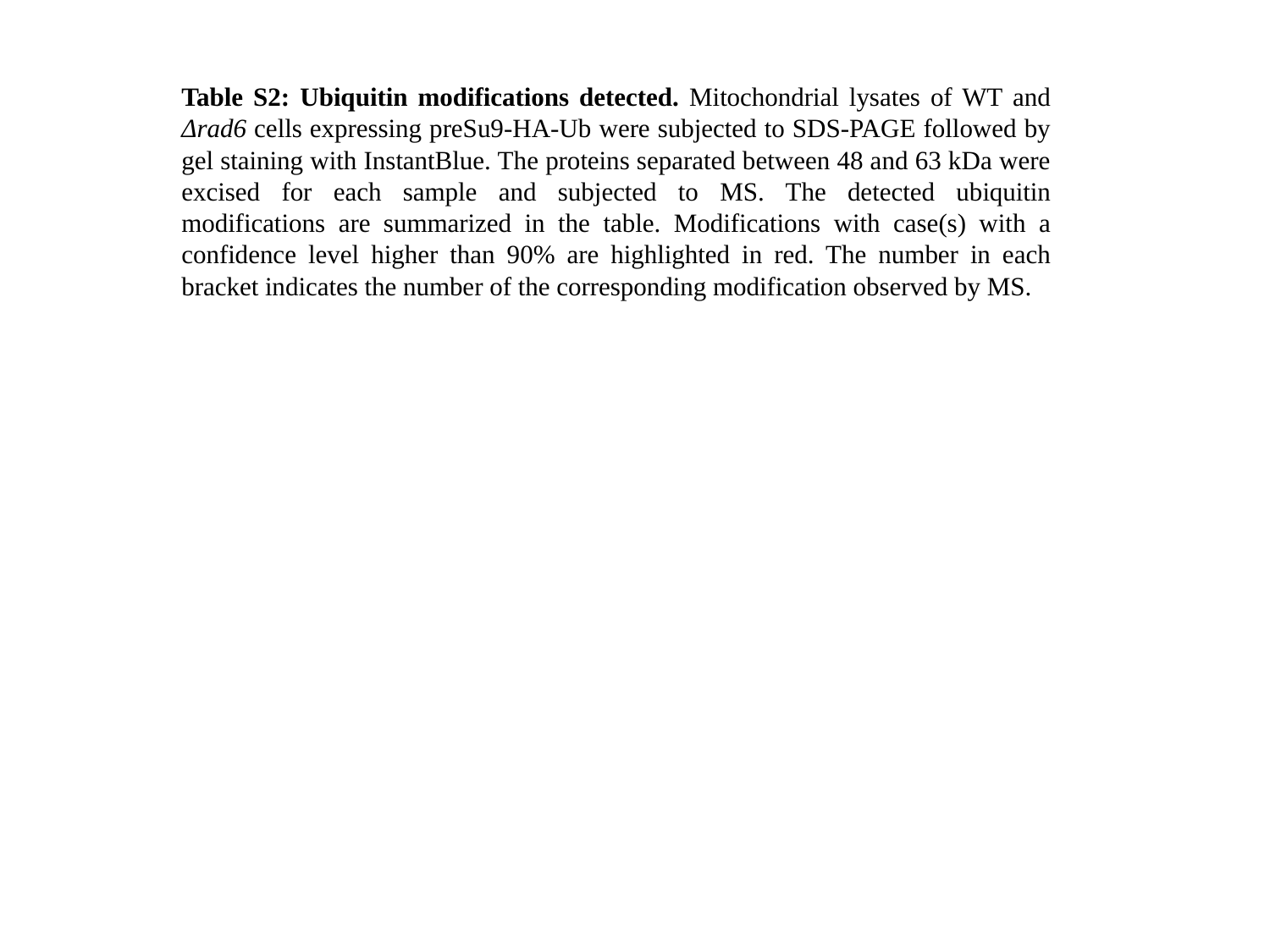

Table S2: Ubiquitin modifications detected. Mitochondrial lysates of WT and Δrad6 cells expressing preSu9-HA-Ub were subjected to SDS-PAGE followed by gel staining with InstantBlue. The proteins separated between 48 and 63 kDa were excised for each sample and subjected to MS. The detected ubiquitin modifications are summarized in the table. Modifications with case(s) with a confidence level higher than 90% are highlighted in red. The number in each bracket indicates the number of the corresponding modification observed by MS.

## Slide 13
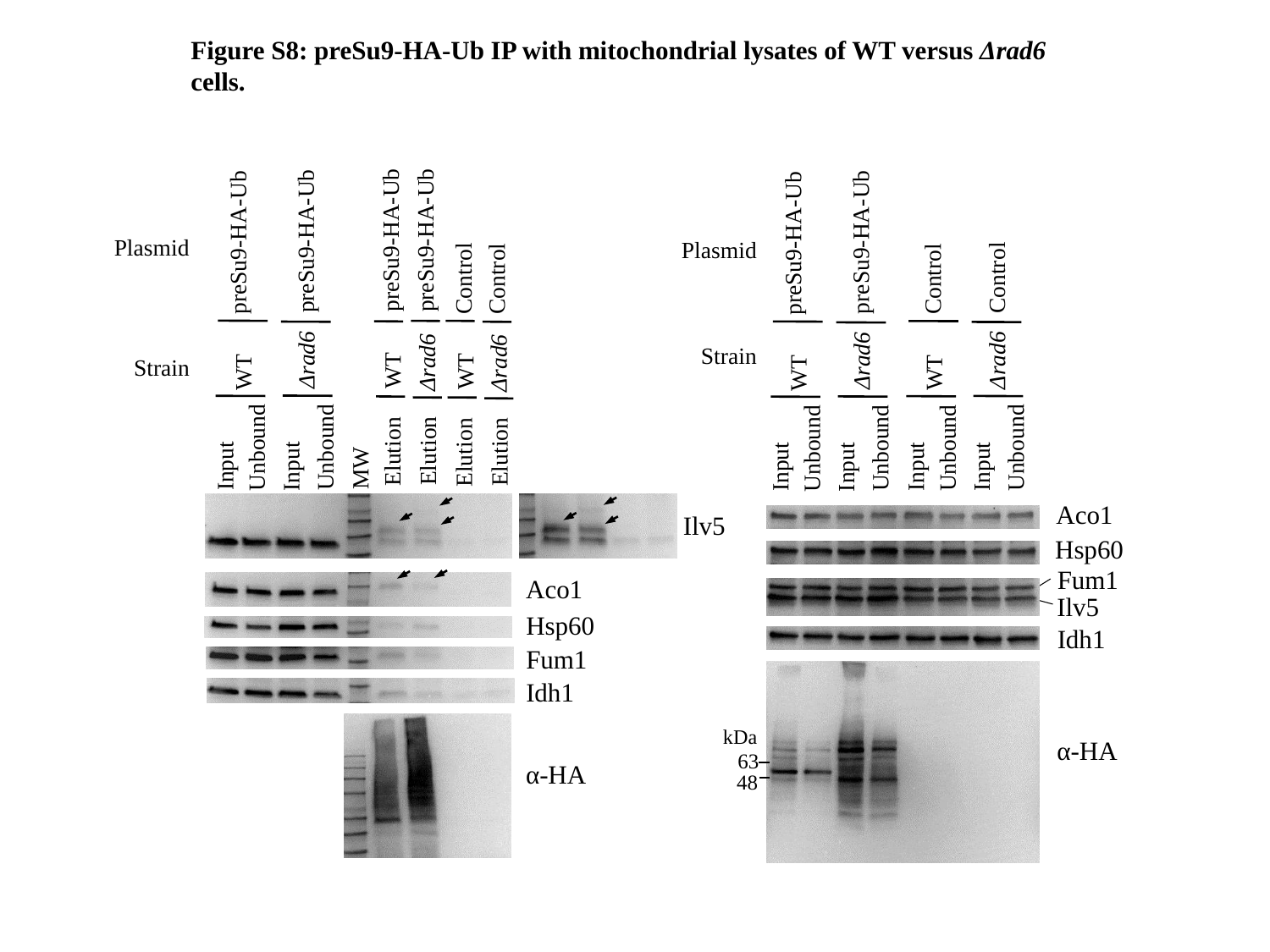

Figure S8: preSu9-HA-Ub IP with mitochondrial lysates of WT versus Δrad6 cells.
preSu9-HA-Ub
preSu9-HA-Ub
preSu9-HA-Ub
preSu9-HA-Ub
preSu9-HA-Ub
preSu9-HA-Ub
Plasmid
Plasmid
Control
Control
Control
Control
Δrad6
Δrad6
Δrad6
Δrad6
Δrad6
Strain
Strain
WT
WT
WT
WT
WT
Unbound
Unbound
Unbound
Unbound
Unbound
Unbound
Elution
Elution
Elution
Elution
MW
Input
Input
Input
Input
Input
Input
Aco1
Ilv5
Hsp60
Fum1
Aco1
Ilv5
Hsp60
Idh1
Fum1
Idh1
kDa
α-HA
63
α-HA
48

## Slide 14
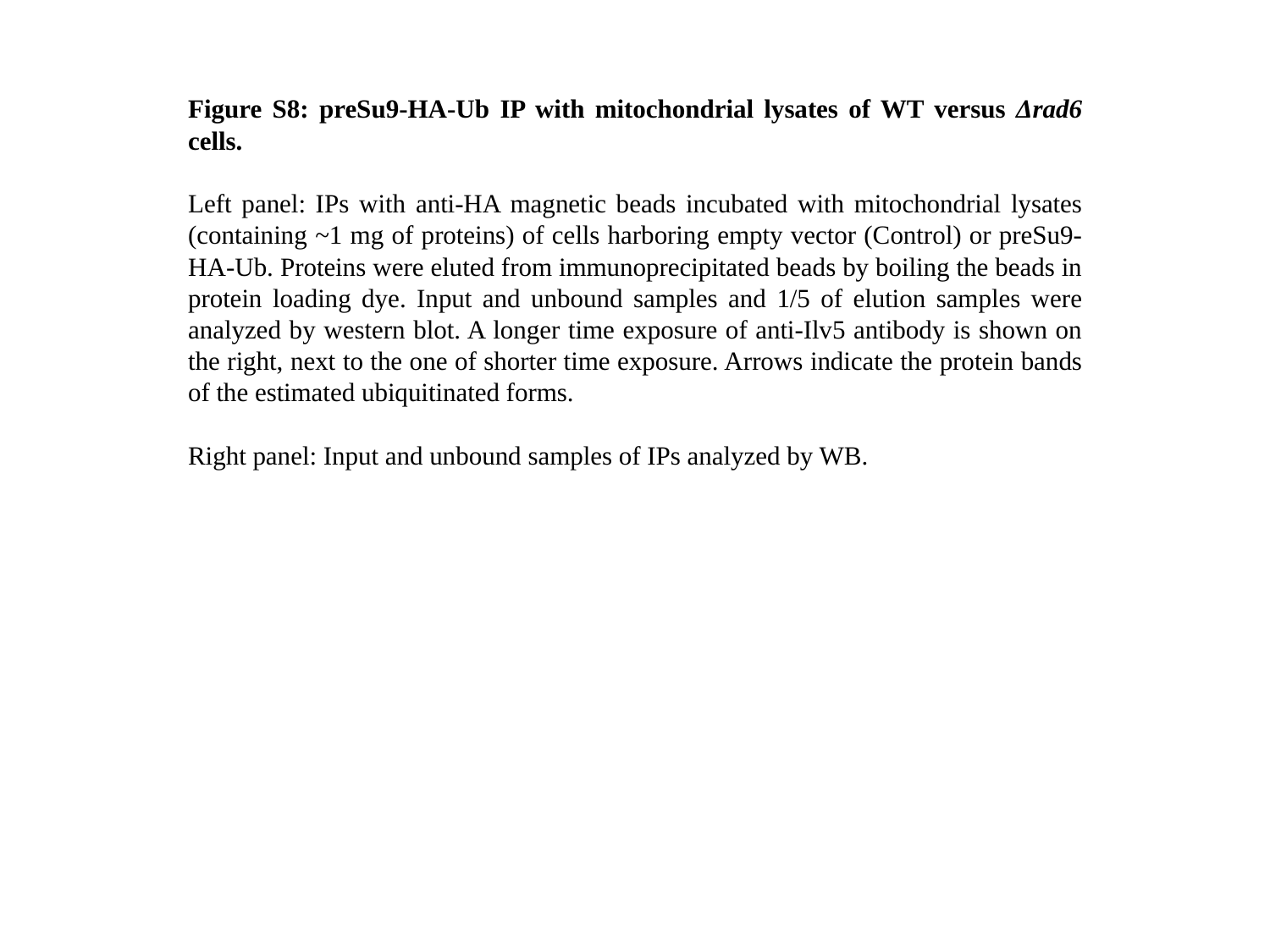

Figure S8: preSu9-HA-Ub IP with mitochondrial lysates of WT versus Δrad6 cells.
Left panel: IPs with anti-HA magnetic beads incubated with mitochondrial lysates (containing ~1 mg of proteins) of cells harboring empty vector (Control) or preSu9-HA-Ub. Proteins were eluted from immunoprecipitated beads by boiling the beads in protein loading dye. Input and unbound samples and 1/5 of elution samples were analyzed by western blot. A longer time exposure of anti-Ilv5 antibody is shown on the right, next to the one of shorter time exposure. Arrows indicate the protein bands of the estimated ubiquitinated forms.
Right panel: Input and unbound samples of IPs analyzed by WB.
